# Extracellular Vesicles: Packages Sent With Complement

**DOI:** 10.3389/fimmu.2018.00721

**Published:** 2018-04-11

**Authors:** Ebru Karasu, Steffen U. Eisenhardt, Julia Harant, Markus Huber-Lang

**Affiliations:** ^1^Institute of Clinical and Experimental Trauma-Immunology, Universitätsklinikum Ulm, Ulm, Germany; ^2^Division of Reconstructive Microsurgery, Department of Plastic and Hand Surgery, University of Freiburg Faculty of Medicine, University of Freiburg Medical Centre, Freiburg, Germany

**Keywords:** extracellular vesicles, exosomes, microvesicles, complement, complement activation, immune response, therapeutic vehicle

## Abstract

Cells communicate with other cells in their microenvironment by transferring lipids, peptides, RNA, and sugars in extracellular vesicles (EVs), thereby also influencing recipient cell functions. Several studies indicate that these vesicles are involved in a variety of critical cellular processes including immune, metabolic, and coagulatory responses and are thereby associated with several inflammatory diseases. Furthermore, EVs also possess anti-inflammatory properties and contribute to immune regulation, thus encouraging an emerging interest in investigating and clarifying mechanistic links between EVs and innate immunity. Current studies indicate complex interactions of the complement system with EVs, with a dramatic influence on local and systemic inflammation. During inflammatory conditions with highly activated complement, including after severe tissue trauma and during sepsis, elevated numbers of EVs were found in the circulation of patients. There is increasing evidence that these shed vesicles contain key complement factors as well as complement regulators on their surface, affecting inflammation and the course of disease. Taken together, interaction of EVs regulates complement activity and contributes to the pro- and anti-inflammatory immune balance. However, the molecular mechanisms behind this interaction remain elusive and require further investigation. The aim of this review is to summarize the limited current knowledge on the crosstalk between complement and EVs. A further aspect is the clinical relevance of EVs with an emphasis on their capacity as potential therapeutic vehicles in the field of translational medicine.

## Background

**Figure 1:**
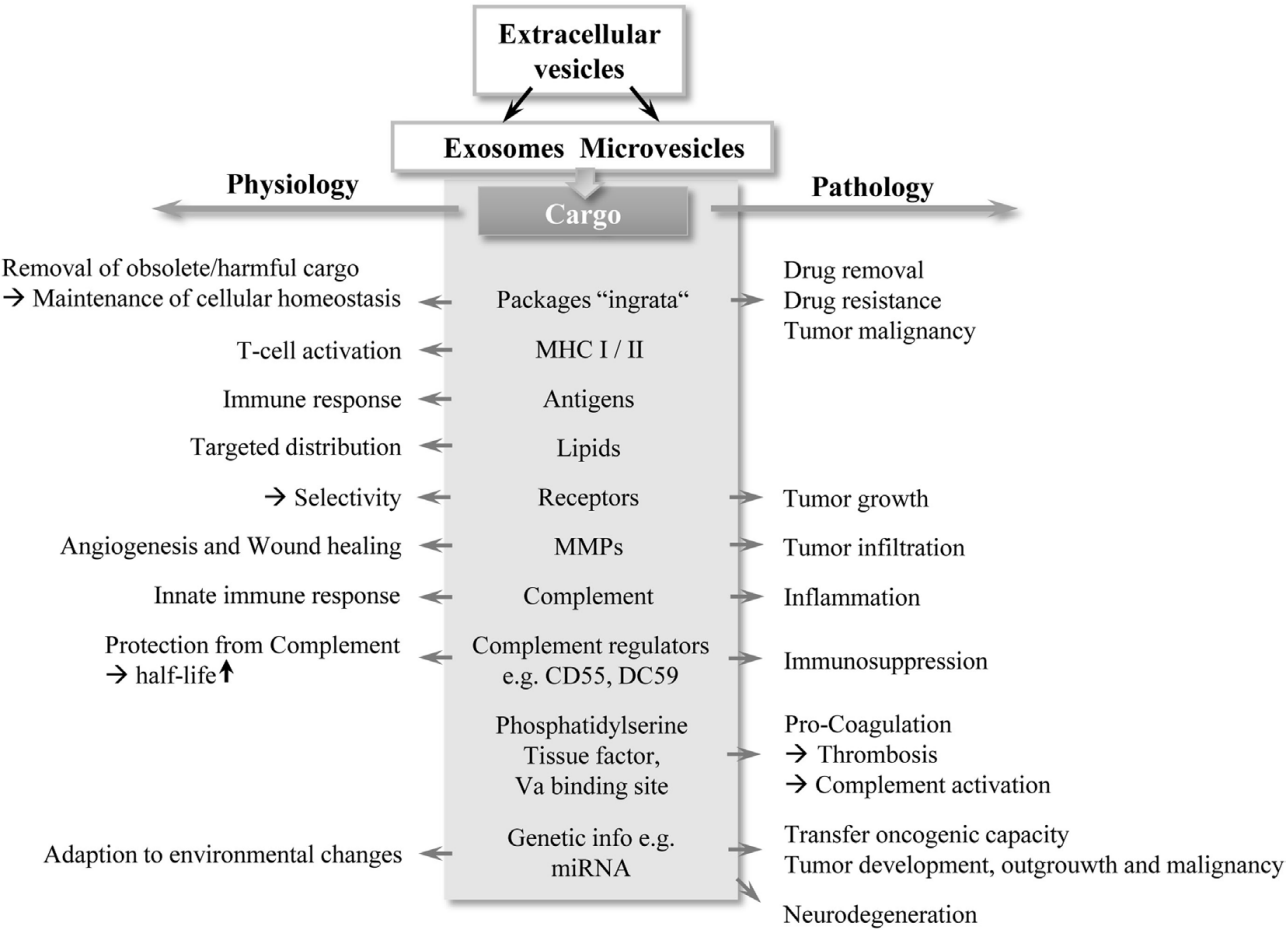
A double-edged sword: schematic overview of EV functions. EVs, in particular exosomes and MVs contain different cargo types, highlighted in gray in the middle section in the figure. Depending on their cargo composition, EVs exert bi-directed functions (depicted by arrows) with important contribution to physiology and pathology. Abbreviations: EV, extracellular vesicles; MHC: major histocompatibility complex; MMP: matrix metalloproteinase; MV, microvesicle.

Extracellular vesicles (EVs) are likely to represent an efficient, robust, and economic manner for exchanging information between cells. Throughout evolution, communication via extracellular cargo carriers appears to be a highly conserved method. This form of cell-to-cell communication is found in lower eukaryotes, bacteria, and plants as well as in higher organisms (1). In addition to cellular communication, EVs are important for survival and adaption to (micro-) environmental changes. For example, bacteria are able to form EVs, termed bacterial outer membrane vesicles (OMV), which contain RNA, DNA, endotoxins, or virulent molecules (2, 3). By shedding OMV with specific cargoes, bacteria influence the behavior and/or property of other bacteria (3). These vesicles play a crucial role in quorum sensing, a known strategy in bacterial colonies to adapt their social group activity to their environment by sending extracellular “packages” (4). Plants are also known to produce EVs, particularly in response to pathogen exposure (5). However, the role of EVs in mammals appears to be more complex. Depending on their content, these packages are capable of influencing a variety of cellular functions and contribute to physiological homeostasis, but also have profound roles in pathological conditions, as illustrated in Figure 1. For example, shedding of endothelial cell-derived vesicles with matrix metalloproteases (MMPs) has been described, which is important for matrix degradation and angiogenesis during wound healing and development, but also during tumor progression (6). Their association with tumors has been extensively investigated. Early studies revealed the involvement of EVs in tumor malignancy by transferring metastatic capacity to other cancer cells (7). Addressing immunity, recent findings suggested that EVs have profound effects on the innate immune system, and consequently are also able to modulate adaptive immunity (8). In particular, the complement system as the first-line of innate immunity represents an important interaction partner for EVs and together they are associated with thrombotic and inflammatory conditions and appear to influence patient morbidity and mortality (9–11). This review focuses on the current state of knowledge on interactions between EVs and complement.

## Extracellular Vesicles

### Morphological Characterization

During programmed cell death, cells are known to release vesicles to the extracellular environment (12). However, the fact that healthy cells also release vesicles to the extracellular environment has only been recently discovered. These cell-derived particles, termed EVs, can be assigned to three main groups: apoptotic bodies, exosomes, and microvesicles (MVs). This classification is based on their characteristic features, including cellular origin and their size, function, composition, and molecular cargo (1).

Apoptotic bodies are released solely from cells undergoing apoptosis (12, 13). Cellular fragmentation occurs with the formation of 1,000–5,000 nm apoptotic blebs, which become packed with intact cellular organelles, DNA, and histones, and subsequently being phagocytized by neighboring live cells without activating an inflammatory or autoimmune response (13–15). By contrast, both exosomes and MVs are released from healthy cells during physiological and pathological conditions.

Exosomes are small spherical vesicles of size 30–120 nm and are produced from late endocytic compartments, known as multivesicular bodies (MVBs) (14). They are produced by inward invagination of endosomal membranes to form MVBs, which subsequently fuse with the plasma membrane and release their intraluminal vesicles as exosomes to the extracellular milieu (16). Because of their endocytic origin, exosomes are commonly enriched in endosome-associated proteins, including Rab GTPases, SNAREs, annexins, and tetraspanins (CD9, CD63, and CD81). Some of these proteins (e.g., the endosome-specific proteins Alix and Tsg101) are normally used as exosome markers (17, 18). However, recent studies identified that exosome-specific markers are also present in MVs (19).

Microvesicles, also referred as ectosomes, microparticles and nanoparticles, directly derive from the cell surface and are larger than exosomes, with a size ranging from 100 to 1,000 nm (20). MVs expose high levels of integrins (21). Although common protein markers used to define these vesicles are selectins, integrins, and the CD40 ligand, less is known about the protein content of MVs (22).

A major and still ongoing challenge is to define methods that will allow discrimination between exosomes and MVs (23, 24). At present, a precise definition and terminology for the characterization of exosomes and MVs remain to be resolved (25). It appears to be difficult to distinguish between these two subgroups when based on characteristics like structure, size, cargo, and protein composition (26). Therefore, there is a broad interest in novel methods of isolation and purification. To further understand the origin of the different vesicle populations and to unravel their (patho-) physiological and pathological relevance, a better mechanistic understanding on their biogenesis and secretion is also required (24).

### Multiple Functions of EVs

#### Cellular Homeostasis

Several studies have demonstrated that eukaryotic vesicles are used to remove obsolete, undesired, or even dangerous cellular molecules or protect the cells and organism from waste or drug, thus contributing to maintenance of cellular homeostasis (1, 26). Interestingly, aged erythrocytes release hemoglobin-containing EVs to enhance clearance of old erythrocytes from the circulation (27). Furthermore, cells are able to remove harmful cytoplasmic DNA in exosomes to maintain cellular homeostasis (28).

#### Transfer of Genetic Information

Recent studies indicated that EVs can contain genetic material such as RNA and noncoding RNAs, including miRNA, and are indispensable for cellular communication and mRNA homeostasis (29). Further studies demonstrated that such EV cargoes could be successfully transferred to recipient cells in culture, leading to functional consequences for the recipient cell (30, 31). Specific miRNAs are enriched in EVs derived from immune cells and thereby mediate proper immune responses or represent important regulators during inflammation (1, 22). However, tumor-derived EVs containing specific miRNAs play profound roles in tumor progression, for example, by supporting immunosuppression or transferring oncogenic capacity (7, 22, 32).

#### Promotion of Coagulation

Although vesicle release has been proposed to be beneficial for the cell, the vesicles can also represent a danger to their environment, for example in blood, where vesicles can provide a surface as a focal point for coagulation activation, resulting finally in thrombus formation (33). Blood coagulation is also triggered by vesicle-mediated interaction between macrophages, neutrophils, and thrombocytes. Thrombocytes shed MVs bearing tissue factor (TF) on their surface, which interact with macrophages, endothelial cells, or further thrombocytes (20, 34). Supporting this, MV-bound TF can be detected in a variety of diseases, including trauma, sepsis, and cancer (35, 36). This so-called “blood-borne” TF may contribute to the development of thrombosis that is associated with these diseases (36–39).

#### Immune Modulation

A sufficient immune response is characterized by a sufficient communication between the innate and the adaptive immune system (40). Immune cell populations from both the innate and adaptive immune systems shed EVs with specific cargo, thus an exchange of EVs appears to be crucially involved in regulation of the immune responses (41). Exosomes released by immune cells have a bi-directional function; they may act as antigen-presenting vesicles, thereby for example stimulating anti-tumoral immune responses, or as inducers of tolerogenic effects suppressing inflammation (8, 42, 43). Early studies showed that immune cell-derived EVs carry MHC class I, MHC class II, and T cell co-stimulatory molecules (43, 44). *In vitro* as well as animal studies demonstrated that antigen-presenting cell (APC)-derived exosomes effectively stimulate T-cell responses, thus they are critical for proper immunological responses (45). Macrophages/monocytes are known to release EVs enriched with the miRNA molecule miR-223, a key regulator of myeloid cell proliferation and differentiation (46). Polymorphonuclear leukocyte (PMN)-derived MVs specifically bind to macrophages/monocytes, dendritic cells (DCs), and endothelial cells and thereby can alter the immune response. Interestingly, immature DCs alter their immunological function in the presence of PMN-derived MVs and consequently feature tolerogenic properties (1).

#### Specificity and Stability

In the human body, EVs can be released from many, if not all, cell types and are abundant in many body fluids, including blood, urine, saliva, cerebrospinal fluid (CSF), ascites, and breast milk (47–55). In this context, it remains unclear, whether shed EVs are addressed for a specific recipient cell, and if so, what causes tissue or organ specificity, their stability and their uptake. Proteomic profiling of EVs identified different types of glycan-binding proteins or glycosylation patterns, which appear to determine the target cells or influence interactions with them (1). For example, B-cell-derived EVs contain α-2,3-linked sialic acid, which can be captured by sialoadhesin (CD169), an adhesion molecule found on macrophage surfaces (56). Because of their variable protein content and composition, EVs have different forms of functionality. Surface-expressed receptors appear to play an essential role in bio-distribution, or binding of EVs to their target cells or to extracellular matrix components (1). Of note, by transferring functionally active receptors, including CCR5, the target-cell phenotype can be modified or even intracellular signaling pathways can be influenced (57). Once released, EVs can bind to neighboring cells or to the extracellular matrix components, or traffic passively through the bloodstream or body fluids (8). The bio-distribution of EVs depends on several aspects, including the parent cell source, availability of recipient cell types, and clearance from the circulation by retention, uptake, and internalization (1). Certain blood-borne EVs are rapidly captured by marginal zone phagocytes of the spleen, liver Kupffer cells, and DCs and macrophages in the lung, resulting in a short half-life in the circulation (44, 58). However, human platelet-derived EVs display a half-life of up to 6 h (59). Furthermore, some EVs express glycosylphosphatidylinositol (GPI)-anchored CD55 and CD59, thereby protecting them from complement-mediated lysis (60). EVs display different lipid compositions of the membrane bilayer in comparison to their parent cells, suggesting a sorting process during EV formation (48). Many studies confirmed a specific lipid sorting, with a predominant content of cholesterol, phosphatidylserine (PS) and sphingolipids present in EVs. Cholesterol appears to be an essential lipid for regulating EV formation and release (38, 61). It has been proposed that EVs enriched with cholesterol and long saturated fatty acids of sphingolipids display a tighter lipid packaging and improved physiochemical features by providing greater stability, structural rigidity, and resistance (1). Established uptake mechanisms include clathrin-mediated endocytosis (CME), phagocytosis, macropinocytosis, and plasma or endosomal membrane fusion, which are reviewed elsewhere (62). Noteworthy, pH appears to modulate uptake capacity by influencing electrostatic charges between EVs and the cell plasma membrane (63).

## EV–Complement System Interaction

### The Complement System

**Figure 2:**
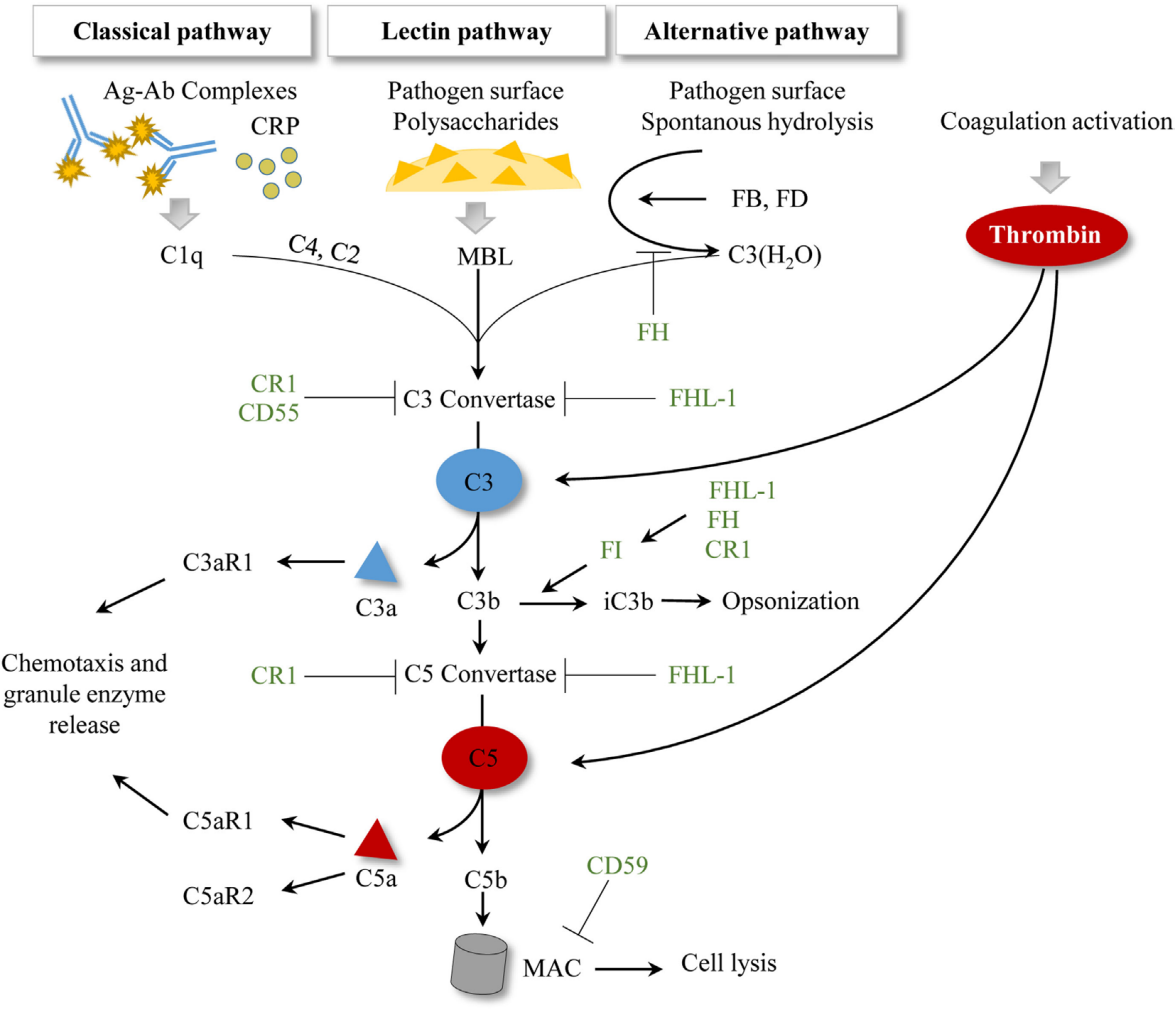
Simplified scheme of established complement activation pathways. To date, three activation pathways for complement have been described. Antibody–antigen complexes or the acute phase protein CRP initiate the classical pathway by allowing C1q binding. The lectin pathway is initiated by MBL binding to pathogen surfaces and to pathogen-specific proteins. The alternative pathway is characterized by spontaneous hydrolysis of C3 to C3(H_2_O), which can in turn bind the plasma proteins FB and FD. All three pathways result in a C3 convertase, which cleaves C3 and subsequently generates C5 convertases and initiates MAC formation. Activation of the coagulation cascade generates thrombin, which can cleave C3 and C5 in a C3-/C5-convertase independent manner. Complement regulatory proteins are represented in green. CR1 facilitates the decay of C3 and C5 convertases and is a cofactor of FI. CD55 accelerates the decay of the C3 convertase. CD59 inhibits the MAC formation. Factor I regulates the processing of C3b to iC3b, where FHL-1, FH, and CR1 show cofactor activities. FH also regulates the formation of the alternative pathway C3 convertase. FHL-1 supports the decay of C3 and C5 convertases. Abbreviations: Ag–Ab, antigen–antibody; CR1, complement receptor 1; CRP, c-reactive protein; FB, factor B; FD, factor D; FH, factor H; FHL-1, factor H-like protein 1; FI, factor I; MAC, membrane attack complex; MBL, mannan-binding lectin.

The complement system represents an important arm of innate immunity and is frequently referred to as the first barrier against pathogens (64). It is well known that the complement cascade consists of more than 30 proteins, which function in a well-orchestrated order of activation and regulation (65). Beyond opsonization and pathogen elimination, this biological system is involved in the regulation of the cytokine/chemokine release, stays in close interaction with the coagulation cascade, and has a crucial impact in tissue inflammation and repair (66). Generally, three pathways for complement activation have been described: the *classical* pathway (CP), the *lectin* pathway, and the *alternative* pathway. All three pathways lead to the generation of a C3 convertase which subsequently cleaves the central molecule C3 and leads to the release of anaphylatoxins C3a and C5a, and activation of the terminal cascade of complement, resulting in the membrane attack complex (MAC) formation with cell lysis (67). The anaphylatoxins can also bind to their respective receptors (C3aR, C5aR1, C5aR2), and mainly exert inflammatory effects, including leukocyte attraction (65, 68). Recently, the serine protease thrombins have been identified to activate the complement system by direct cleavage of C3 and C5 (65). A simplified overview of the complement system is presented in Figure 2.

### EVs and Complement As Interaction Partners

Higher numbers of circulating vesicles are found in local and systemic inflammation, which is also characterized by the appearance of complement activation products (10, 36). Their co-presence suggests a link between the complement system and EVs, which may influence innate as well as adaptive immune responses. To date, literature in this research field is limited and warrants further experimental investigation. Nevertheless, several studies provide increasing evidence supporting an important crosstalk between these biological systems. On the cellular level, endothelial cells, PMNs, monocytes, erythrocytes, and platelets appear to be mainly involved (9, 11, 42, 69). For both systems, it has been shown that activation and interaction of the complement system with other cascades such as coagulation and MVs lead to both pro-inflammatory and anti-inflammatory directions and can also affect morbidity and mortality in diseases, including the setting of trauma and sepsis (10, 36).

#### Complement Evasion Through EV Shedding

The first description of vesicle–complement interaction focused on the release of vesicles directly from the cell membrane (ectocytosis) upon stimulation of neutrophils with sublytic amounts of the MAC (70). After complement attack, a large number of membrane protrusions appear on the cellular surface following vesicle shedding from the cells (10, 71). In accordance with this, PMNs activated with either N-formyl-methionyl-leucyl-phenylalanine (fMLP) or C5a release vesicles from their membrane within minutes, which overall exhibit features corresponding to MVs (72).

Strikingly, various cells are able to evade complement attack by allowing the removal of the MAC from the cell surface by a calcium-dependent elimination process (73–75). Several studies demonstrated that MAC clearance occurred actively and rapidly from the plasma membrane, assuring cell survival and recovery from complement attack. Such complement-induced MV shedding was reported in erythrocytes, PMNs, glomerular epithelial cells, and tumor cell lines, including U937 and K562 (73, 74, 76, 77).

Moreover, cytotoxic effects were found on oligodendrocytes after complement activation with resultant C9 formation, which in turn release MAC-enriched vesicles from their surface (78, 79). In support of this, identical MAC-containing vesicles were detected in the CSF of multiple sclerosis patients, indicating that complement-mediated injury contributes to myelin damage *in vivo*, where vesicle shedding appears as an important regulatory process (79).

#### EVs Modulate Immune Responses *via* Interaction With Complement Regulators

In addition to protection from complement attack, EVs are key players in the regulation of immunological responses. APCs interact with immune cells, particularly T-cells, by shedding exosomes with T-cell stimulatory properties (8). However, exosomes, derived from antigen-processing compartments, are likely to be associated with antigenic proteins, and thereby are particularly prone to bind immunoglobulins (60, 80). These in turn activate the *classical* pathway (CP) resulting in rapid opsonization and lysis. Remarkably, even non-antigen harboring exosomes are described to activate the complement cascade, where C1q plays an important role by simply binding to lipid membranes through electrostatic interactions rather than in an antibody-dependent manner (81, 82). However, exosomes derived from APCs also appear capable of escaping from complement-mediated lysis. Interestingly, these exosomes express the GPI-anchored complement regulators CD55 and CD59 (60). CD55 regulates the C3 and C5 convertases, whereas CD59 inhibits the formation of MAC (83). These findings strongly indicate that exosomes equipped with complement regulators like CD55 and CD59 are able to escape complement attack, which contributes to their stability and longer circulatory availability (84). These vesicle-bound complement regulators may be important for regulation of the immune response by stimulating/inhibiting T-cell responses as well as cross-priming with other immune cells (60).

A further member of the complement regulators, complement receptor 1 (CR1) also interacts with EVs. PMN-derived MVs are described to express clusters of CR1, which allow them to bind efficiently to opsonized bacteria. Interestingly, of the other complement regulatory proteins expressed by the PMN, only CD59 co-localized with CR1, whereas CD55 was almost absent (72). Of note, in the absence of complement molecules, PMN-derived MVs preferentially bind to endothelial cells, whereas in the presence of complement, adhesive features of PMN-derived MVs change: PMN-derived MVs can bind C1q, and subsequently C3, and are thereby capable of binding to erythrocytes via CR1 (85). By binding to erythrocytes, these opsonized MVs may resemble circulating immune complexes, which are transported to the liver and spleen and are cleared rapidly from the circulation. Consequently, their adherence to erythrocytes may prevent binding to endothelial cells, thus leading to reduced bio-availability for interactions with other immune cells and avoiding harmful fixation in tissues (86, 87). Although speculative, binding of MVs to erythrocytes may also result in altered erythrocyte function. Aged erythrocytes shed MVs enriched with CR1 and CD59 (88). Therefore, old erythrocytes may no longer be protected from opsonization and complement attack, which enhances their removal from the circulation by phagocytosis. Overall, the release of cellular complement regulators via MVs appears to be important during physiological but also pathological conditions. The presence of complement regulators on EVs was also described in the context of age-related macular degeneration (AMD). AMD is characterized by a complement-mediated inflammation in the macula leading to cellular damage and vision loss. Retinal pigmented epithelium (RPE) cells possess immunosuppressive features and modulate monocyte activity through EVs. Furthermore, RPE-derived EVs appear to regulate complement activity by interacting with complement regulators. Under inflammatory conditions, membrane complement regulators including CD55, CD59 and CD46 are present on RPE-derived EVs. As a consequence, cells are vulnerable to a complement attack, which can lead to cellular death (89). Additionally, mutations in complement regulator genes, including the CFH gene encoding for FH and FHL-1 are involved in the pathology of AMD (90–92). Besides C3 binding, RPE-derived EVs are able to bind FH, suggesting a regulatory function (93). Mutations in the CFH gene may impair the binding of FH and FHL-1 to EVs. Theoretically, in the absence of FH/FHL-1, C3-coated EVs may be recognized as immunological complexes by invading immune cells, and thus may support the inflammatory process in AMD.

#### EV-Mediated Immunosuppression *via* C5aR1 Shedding

Frequently, immunosuppression is a consequence of multiple trauma, hemorrhagic shock, or progressive sepsis, leading to impaired immune function by development of complementopathy and coagulopathy with a fatal clinical outcome (94–96). Rapid complement activation and exhaustion after severe tissue trauma lead to complement dysregulation (94). In clinical sepsis, an enhanced presence of the complement C5a receptor C5aR1 on MVs in patients with septic shock has been demonstrated, suggesting an important role in the outcome. In addition, MV shedding with C5aR1 negatively correlated with survival in sepsis patients (10). In a cohort of sepsis patients, neutrophils exhibit decreased C5aR1 expression, but enhanced numbers of circulating C5aR1 (C5aR1 on MVs), resulting in an impairment of neutrophil function. Sera from non-survivors after septic shock contained much more circulating C5aR1 than in sera from survivors. However, it cannot be excluded that the circulating C5aR1 was also bound to membrane fragments other than MVs. Nevertheless, *in vitro* experiments with human neutrophils demonstrated that treatment with C5a or the acute phase-protein C-reactive-protein (CRP)—both of which are significantly enhanced in plasma during septic shock—resulted in an almost complete loss of C5aR1 on neutrophils, and significantly increased numbers of MVs carrying C5aR1 on their surface leading to an acquired dysfunction of neutrophils (10). Furthermore, in a rodent sepsis model, circulating C5aR1 was co-expressed with a granulocyte-specific MV marker CD66e, confirming microvesicle-mediated shedding of C5aR1 (10). In support of this, a further study identified a 20-fold higher gene expression of C5aR1 in blood leukocytes of 26 patients with severe sepsis compared to healthy volunteers (97). This finding may be explained by a cellular compensatory mechanism, by upregulating *de novo* synthesis of C5aR1 due to enhanced C5aR1 shedding on microvesicles.

#### EVs As a Platform for Complement Activation

In addition to regulatory functions, EV interactions with complement may aggravate the complement response. Several studies revealed increased numbers of circulating MVs in both thrombotic and inflammatory diseases, where complement activation is also present (98). In synovial fluid of rheumatoid arthritis patients, significantly increased levels of leukocyte-derived MVs were detected with complement components bound on their surface, including C1q, C4, and C3 (11). *In vitro* studies provided further evidence by demonstrating that leukocyte-derived MVs can bind C1q and activate the CP, subsequently leading to deposition of C4 and C3 (70, 75, 76). In accordance, Gasser and Schifferli demonstrated that PMN-derived MVs could activate complement. This activation was mediated by the CP, and only after C1q deposition on MVs, C4 and C3 fragment fixation did occur (85). Further downstream, activation of MAC formation occurred on the surface of MVs (70). In agreement with this, human erythrocyte-derived MVs were reported to fix C1q, which was followed by activation of the CP with binding of C3 fragments (99). As mentioned above, elevated numbers of circulating EVs (particularly MVs) are present during inflammatory conditions, which are generally associated with enhanced complement activation (100). In conclusion, cells may primarily compensate overwhelming complement activation by releasing more MVs. These MVs may act as scavengers by fixing complement molecules and allowing MAC formation on the MV surfaces, thereby protecting the parent cells and tissues from complement attack.

C-reactive protein (CRP) was also described as an important mediator for complement binding on MV surfaces. CRP is synthesized in the liver in a complement-dependent manner in its native pentameric form (pCRP) (101). According to the current findings, pCRP localizes to injured tissues and undergoes conformational changes, leading to the formation of pCRP*, which still maintains the symmetry of pCRP, but is detected by a neoepitope-specific antibody, and/or can even dissociate into neoepitope-expressing monomeric CRP (mCRP) (102). Studies indicate that both pCRP* and mCRP aggravate the inflammatory response by complement activation, but currently, it is impossible to distinguish between pCRP* and mCRP (102–104). Interestingly, it was also demonstrated that binding pCRP on MV surfaces induced the generation of pCRP*/mCRP, which in turn can activate the complement system (102). The authors demonstrated that MVs with bound CRP could fix C1q, thus activating the CP. This study could detect significantly higher levels of CRP bound on MVs in patients after myocardial infarction compared to healthy controls. Additionally, Braig et al. analyzed the presence of native CRP (pCRP) and neo-epitope-expressing CRP (pCRP*/mCRP) on MVs of different cellular origins (leukocytes, platelets, and activated platelets), which confirmed findings of Habersberger et al. and suggests a diagnostic use in this clinical setting (102, 105). In all cellular populations, a significantly higher deposition of both pCRP and pCRP*/mCRP was detectable on the respective MVs (102). *In vitro* studies revealed a significantly elevated deposition of the C3 cleavage product C3b and iC3b on MVs in the presence of pCRP*, suggesting a much more complex interaction mechanism with the AP. In contrast to C3b, iC3b is not able to form an AP C3 convertase and thus functions rather anti-inflammatory or tolerogenic (106–108). Furthermore, surface bound iC3b is a potent oposonin, interacts with receptors, and stimulates phagocytosis and antigen presentation to cells of the adaptive immune system (109). Factor I and the cofactors FH, FHL-1, and CR1 are known to process C3b into its inactive form iC3b (Figure 2). So far, the mechanisms behind the observed C3b and iC3b presence on MVs remain elusive. Furthermore, it is not clear how CRP is involved in this process. On the one hand, the presence of pCRP* on MVs may enhance the extent of complement activity by recruiting factor B and factor D, resulting in C3b formation. On the other hand, MVs bound with pCRP*/mCRP may recruit FI, FH, and/or FHL-1, which in turn processes the bound or circulating C3b to iC3b. Interestingly, a current study revealed that the complement regulator FHL-1 is able to bind pCRP as well as mCRP, and FH is able to bind mCRP (110). Since pCRP* also expresses a neoepitope, it can be speculated, that pCRP/pCRP* and/or mCRP, which are bound on MVs support FH and FHL-1 recruitment, which in turn enhances the processing of C3b to iC3b. However, even in the absence of CRP, C3 and iC3b are bound on MVs, assuming a further and CRP-independent mechanism (102, 111). It may be possible that C3-coated MVs resemble opsonized pathogen surfaces, resulting in recruitment of FB and FD and in forming an AP C3 convertase. Another explanation include that hydrolyzed C3, C3(H_2_O), is generated and/or bound on MV surfaces, where it may also form an AP C3 convertase. The formed AP C3 convertase may cleave other circulating C3 into C3b and causes its deposition on MVs. Subsequently, EVs may recruit or bind FH, FHL-1, and FI to process C3b to iC3b. However, it is still probable that C3b and iC3b may be generated through the AP without MV involvement, thus MVs only provide a surface for the cleaved products C3b and iC3b. Therefore, further investigations are necessary to clarify the presence of C3, C3b, and iC3b on MVs.

#### EV-Interaction With the Coagulation System Activates Complement

Blood coagulation is known to exhibit extensive crosstalk with the complement system. In particular, activation complement system includes the involvement of the coagulation molecule and serine protease thrombin, which is able to directly cleave both C3 and C5 (Figure 2). Independent of their cellular origin, MVs harbor PS on their surface. *In vitro* studies revealed that these vesicles have pro-coagulant properties and PS represents a platform for thrombin generation thereby also activating complement (112). Furthermore, erythrocyte-derived MVs are described to activate complement via a thrombin-dependent mechanism (69).

Additionally, endothelial cell-derived MVs appear to play an important role in coagulation-mediated complement activation. It is known that endothelial cells express complement factors and regulators as well as receptors on their surface (113). Particularly during infectious or inflammatory conditions, endothelial cells revealed a deposition of complement molecules on their surface (114). Consequently, the endothelium is able to mediate MV shedding after complement activation (9). MAC formation on endothelial cells was reported to be followed by the shedding of MVs with MAC on their surface and of note, additionally expressing binding sites for the clotting factor Va and thus support prothrombinase activity (115). The prothrombinase complex includes Va, Xa, and prothrombin; activation of the complex can lead to thrombin formation, which mediates clotting as well as complement activation (116, 117). Furthermore, blood coagulation can be activated by the release of TF-coated MVs (20). TF is a known key molecule for coagulation and thrombosis initiation (118). TF-coated vesicles have been described to interact with macrophages, endothelial cells, and thrombocytes (20). Therefore, it can be speculated that in addition to blood coagulation, TF-coated MVs are able to initiate complement activation. Although TF alone is unable to directly interact with C3 or C5, formation of thrombin is triggered when membrane-localized TF is exposed to blood (119, 120). These blood-borne TF-MVs may be involved in thrombin formation (38). In this context, thrombin as a potent serine protease can directly cleave C3 and C5 (117).

**Figure 3:**
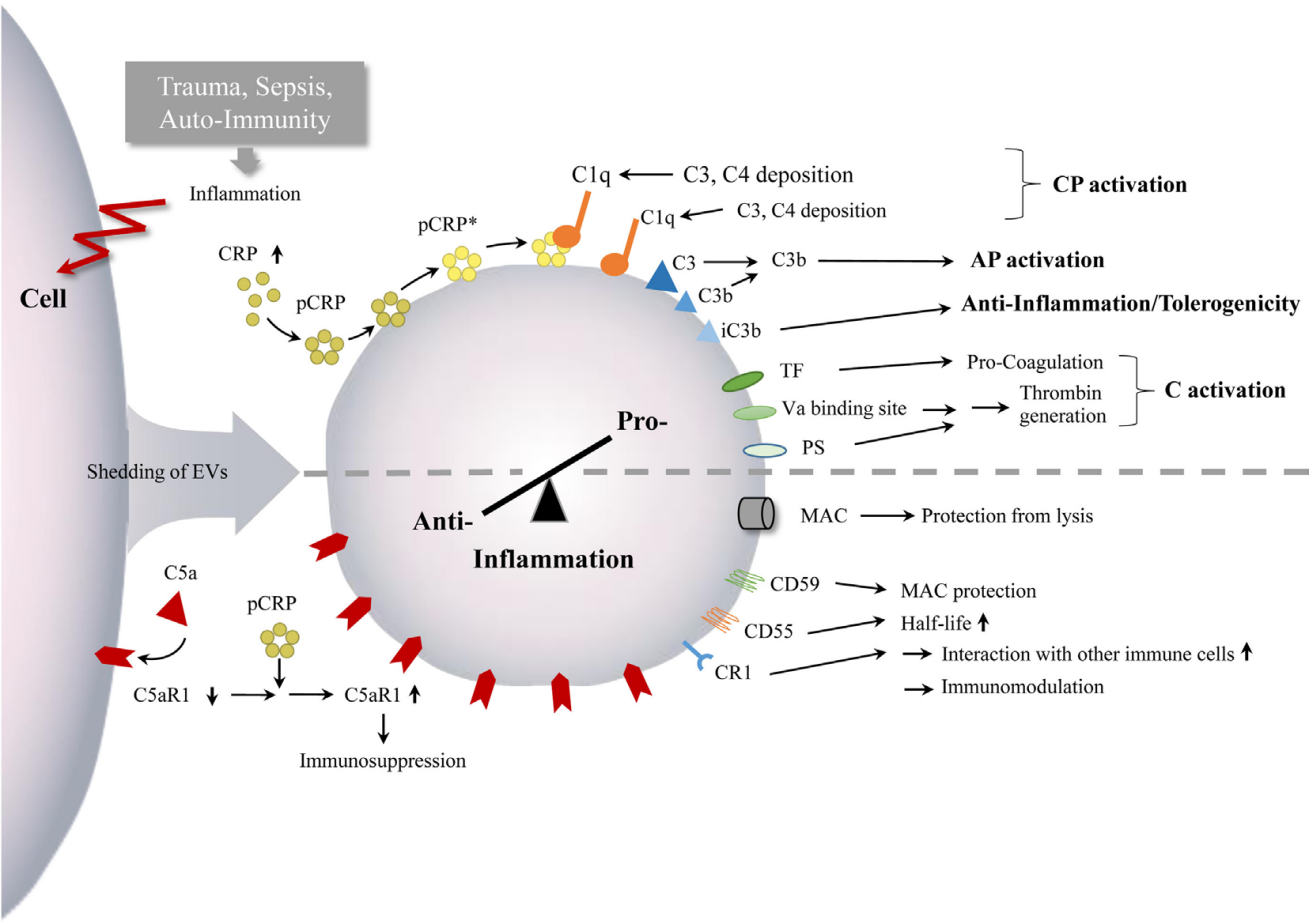
Interaction between EVs and complement. Inflammatory conditions, after trauma, during sepsis as well as in auto-immunity, can lead to enhanced numbers of circulating EVs with complement components. The EVs appear to mediate both complement activation and inhibition, which can monitor or ship the pro-anti-inflammatory immune balance and inflammatory processes, indicated by the semi-dotted line. Abbreviations: AP, alternative pathway; C, complement; CP, classical pathway; CR1, complement receptor 1; CRP, C - reactive protein; mCRP, monomeric CRP; pCRP, pentameric CRP, pCRP*, pentameric CRP, expressing neoepitope of mCRP; PS, phosphatidylserine; TF, tissue factor.

Taken together, current studies provide evidence that complement can promote EV shedding and activation and vice versa. Their crosstalk appears to exert crucial effects on regulating the extent of complement activity and thus modulating both the innate and adaptive immune responses (Figure 3).

## Clinical Relevance

### EVs in Diseases

#### Oncologic Diseases

Early studies indicate a strong contribution of EVs in tumor outgrowth and progression (7). One major known mechanism of EV contribution in this process is mediated by transferring oncogenic activity to other cancer cells. Glioma cells expressing epidermal growth factor receptor variant III (EGFRvIII) secrete MVs harboring EGFRvIII and transfer this receptor to EGFRvIII-negative cancer cells within the same primary tumor, resulting in tumor growth (121). Furthermore, pH is an important factor for EV uptake into recipient cells (63). An increased fusion efficiency of tumor-derived exosomes was identified in cells of metastatic origin in comparison to those derived from primary tumors or normal cells (63). Strikingly, the responsible mechanism for enhanced fusion efficacy was mediated by an acid pH in the tumor microenvironment, which influences rigidity, fluidity, and lipid composition resulting in direct plasma membrane fusion of EVs with enhanced EV trafficking between tumor cells (63). In the view of the tumor, EVs can also function as packages “ingrata”, where they expel therapeutics out of the cancer cell by MV shedding mechanisms (122).

Established mechanisms for tumor progression include degradation of extracellular matrix components, resulting in barrier dysfunction, tumor invasion, and tumor cell migration (123, 124). In this regard, tumor-derived EVs harbor matrix-degradation proteases including matrix metalloproteinase-2 (MMP-2) and MMP-9, which correlated with tumor progression (125, 126). Furthermore, tumor cells have been shown to exploit EVs to contribute to their progression by inactivating T lymphocytes or natural killer cells as well as promoting differentiation of regulatory T lymphocytes to suppress immune reactions (127).

In summary, the main known mechanisms of EVs provide degradation of extracellular matrix components, transfer of oncogenic activity to other cancer cells, and drug resistance (7, 121, 122).

#### Inflammatory Diseases

In the context of inflammation, EVs have been shown to exert both pro- and anti-inflammatory effects and can influence the course of disease. In particular, MVs can not only modulate inflammation but also coagulation. MV production dramatically increases after severe trauma, including traumatic brain injury (TBI), major burn injury, and during sepsis (128–130). In regard of severe sepsis, complement is known to negatively influence the immune response, which can even lead to multiorgan failure (131). Non-survivors of septic shock for example, exhibit increased numbers of MVs bearing C5aR in comparison with sepsis survivors (10). Simultaneously, C5aR expression on neutrophils completely mirrored the expression pattern on MVs, displaying a loss of C5aR on neutrophils in patients who did not survive the lethal consequences of sepsis (10).

Noteworthy, EVs also play critical roles in TBI (132). Similar to other injuries, cell–cell communication is critical for regulating the immune response in TBI (133). TBI features a post-inflammatory immune response, which results in the activation and migration of resident glia and of recruited peripheral immune cells to the injury site (134). Kumar et al. recently found that microglial-derived MVs are responsible for neuroinflammation after TBI (135). In a rodent TBI model, EVs were isolated after injury and revealed an altered expression pattern of miRNA. Expression of miR-212 in EVs decreased, while miR-21, miR-146, miR-7a, and miR-7b were significantly increased after injury, indicating an enhancement loop of neuroinflammation caused by EVs (136).

Extracellular vesicles also modulate ocular immune functions in health and in disease, and they are associated with inflammatory conditions in AMD (137). Additionally, dysregulation of the alternative pathway with consequent over-activation of complement critically influences the pathophysiology of AMD (90, 138). RPE-derived exosomes appear as contributors of inflammation by interacting with complement. Aged mouse RPE cells and human RPE cell lines shed exosomes containing C3 and FH on their surfaces (93). Additionally, the complement regulators CD55 and CD59 were detected on RPE-derived EVs, enhancing complement attack of the parent cells (89). However, it is still unclear how RPE-derived EVs modulate AMD-related complement responses. Overall, EVs represent a highly promising research area not only for the field of ocular diseases. In future approaches, EVs from other ocular cell populations should be analyzed for their contribution to inflammation. For example, corneal fibroblasts are already described to release exosomes, which contain components of the extracellular matrix, which are important for corneal wound healing (139).

#### Neurodegenerative Disorders

Extracellular vesicles inhere multiple and crucial functions in the central nervous system (CNS), including intercellular communication, maintenance of myelination, synaptic plasticity, antigen presentation, and trophic support of neurons, which are reviewed in detail elsewhere (140). It is unsurprising that EVs are associated with amyotrophic lateral sclerosis (ALS), Alzheimer’s disease or Parkinson’s disease, where they can act as carriers of misfolded and neurodegenerative-specific proteins. For example, TAR DNA binding protein (TDP-43) is one of the main pathogenic proteins contributing to ALS (141). Ubiquitinated and hyperphosphorylated abnormal TDP-43 fragments are described to accumulate as vesicle inclusions within neurons and glia, causing cellular death (141, 142). Interestingly, TDP-43-enriched EVs could be extracted from ALS patients, suggesting a crucial impact on the course of disease (143). A detailed account of these findings as well as others, considering the role of EVs in neurodegeneration, is highlighted in a comprehensive review (144). Regarding the role of complement, investigations so far are lacking that link EVs with complement in neurodegenerative disorders.

### EVs As Potential Biomarkers

Extracellular vesicles represent multifunctional entities that appear to play an active role in many biological processes, including the transfer of bioactive molecules between cells and tissues, and the transfer of viruses and prions (145, 146).

Extracellular vesicles, including exosomes and MVs, have promising properties as minimally invasive and novel source of biomaterial in molecular diagnostics (147). Furthermore, EVs can be readily identified and collected from various biological fluids. EVs carry a variety of macromolecules, including DNA, RNA, mRNA, miRNA, and other non-coding RNAs, as well as complement, providing promising targets for disease detection, characterization, monitoring, and therapy. Clinically, circulating exosomes and MVs isolated from cancer patients have been associated with metastasis or relapse, and thus could serve as important diagnostic and prognostic markers as well as therapeutic targets (148). Furthermore, tumor-derived MVs have been described to contain tumor-gene transcripts, which could be used as “liquid biopsies” in the field of clinical diagnostic (149).

In the context of neurodegenerative diseases, there remains a lack of biomarkers, particularly neurodegenerative-specific biomarkers are difficult to detect in CSF (150). Because CNS-derived EVs are described, the fact that they protect their cargo from degradation makes them promising candidates as biomarkers (1). As an additional advantage, the cellular origin can be identified according to surface marker expression profiles (151). Thereby, EVs may help to deliver more knowledge about the cellular and pathogenic processes in specific CNS cell populations. MVs spiked with complement may represent attractive biomarkers to analyze the inflammatory state in patients, including C5aR, which on MVs correlates with the severity of sepsis outcome (10). However, no standardized method for EV isolation and characterization is established in the clinical application (152).

### EVs As Therapeutic Vehicles

In the field of translational medicine, EVs represent promising candidates for therapeutic applications, for example, as vehicles for horizontal molecule transfer. Additionally, they inhere a myriad of potential clinical applications, ranging from oncological destruction to tissue repair and regeneration (42, 153).

Because vesicles can pass the blood-brain barrier (BBB), they could even be considered as naturally occurring liposomes (154). This aspect is of immense importance for pathologies concerning the CNS, including TBI, neurodegeneration and brain cancer, where the BBB dramatically limits drug delivery (155).

As the natural carrier of signal molecules, EVs possess many other favorable properties including stability, biocompatibility, biological barrier penetration, low toxicity, which make them an attractive vehicle for therapeutic delivery (156). Moreover, exosomes may be less immunogenic, less cytotoxic, and non-mutagenic compared to other existing viral-based or liposome-based gene delivery vehicles (157). These characteristics suggest that exosomes can be developed as an ideal vehicle for therapeutic delivery. Because EVs use native mechanisms for cellular entry, internalization and trafficking, therapeutics in the form of small RNA could benefit from EV delivery, including miRNAs, anti-inflammatory agents, and anti-cancer drugs. Tumor acidity is a key contributor to chemo-resistance by protonation and subsequent neutralization in the acidic microenvironment (63). Drug transfer by EVs may overcome this situation and could facilitate an efficient transport of the drug into the tumor cells. To date, the feasibility of EV-based therapies has not been evaluated in clinical trials because there are some major limitations for therapeutic application.

Extracellular vesicles, particularly MVs, contain PS on their surfaces, initiating complement activation and subsequent rapid clearance. Thus, introducing modification of vesicles appear to be inevitable but also therapeutically promising (69). In this context, the design of EVs harboring complement regulators, including CR1 or CD55 as well as CD59, might protect them against complement activation and attack, and thereby prolong their half-life in the circulation and ensure a wide-range systemic distribution, all of which may be of advantage in the treatment of systemic inflammatory conditions. However, PS is also known to induce thrombosis by activating the coagulation cascade, resulting in adverse thrombotic events after clinical application (94). In this case, myristoylated alanine-rich C-kinase substrate (MARCKS) appears to be a promising option, because *in vitro* studies have demonstrated that MARCKS can sense PS and inhibit its functions, and thus may act as an anticoagulant agent (158). Therefore, it would be promising to investigate whether MARCKS is able to inhibit complement activation and/or thrombosis. In addition to cargo delivery, this finding is also of importance for targeting PS-containing MVs, which are for example present in circulating cancer-derived EVs from various cancers and can induce thrombotic events (159).

Unmodified EVs are prone to accumulation, which preferentially occurs in solid organs, including the liver, kidney, and spleen, and consequently results in rapid clearance by bile excretion, renal filtration, or phagocytosis. Therefore, the therapeutic cargoes within EVs might reveal a minimal accumulation in the intended tissue or organs and may instead be enriched in un-intended tissues (160, 161). This bio-distribution profile and off-target effects also disqualify unmodified exosomes from clinically acceptable therapeutics. Though, EVs need modifications to improve their physiochemical properties and therapeutic efficiency. PEGylation of EVs has been analyzed in mice, where they displayed improved cell specificity and prolonged circulation times (162). Nevertheless, PEGylated EVs are still subject to rapid clearance as a result of systemic immunogenicity (163). How EVs target recipient cells *in vivo* remains largely unknown. However, exosomes are selectively enriched in some transmembrane proteins that can be genetically engineered to display ligands/homing peptides on their surface, which confers exosome binding specificity to cells bearing the related receptors (164). Innovative technologies, including phage display and *in vivo* biopanning helped to discover many peptides homing to diseased tissues or organs, providing a potential use for targeted EV-therapy (164). Furthermore, lipid composition also plays an important role for favorable physiochemical properties, influencing vesicle uptake, bio-distribution, and half-life in the organism (1, 23). Therefore, this aspect should be considered for vehicle engineering and may be important for the improvement of liposomal drug delivery systems (161). However, therapeutic application of liposomal drug delivery systems are known to be able to induce a complement activation related pseudoallergy (CARPA), hampering their clinical use (165). The extent of CARPA by liposomes depends on several characteristics, including charge, size, and cholesterol content (166). In contrast to liposomes, EVs have conserved membranous structures, including natural corona, lipid composition, (trans-) membrane proteins, receptors as well as uptake and fusion modalities, which are comparable to those in cells (165). Thus, EVs seem to overcome the CARPA limitations caused by liposomes or nanoparticle-based delivery systems and represent highly promising therapeutic carriers (167).

In the context of inflammation, a noninvasive application of EVs containing anti-inflammatory drugs resulted in a significant inhibition of lipopolysaccharide (LPS)-induced brain inflammation in rodents (168). In a pioneering study, the use of nitrogen cavitation effectively generated a large number of EVs from neutrophils. Strikingly, this artificially generated EVs displayed more favorable properties compared to normal neutrophil-derived EVs, for example could also deliver an anti-inflammatory drug, which successfully showed beneficial effects in acute lung inflammation/injury and in LPS-induced sepsis (169). Hypothetically, such EVs could be used for therapeutic targeting of complement, by engineering EVs with specific binding sites for complement molecules to scavenge excessive soluble complement factors from the blood circulation and protect the host from overwhelming complement activation, for example in the clinical setting of severe trauma or sepsis. Furthermore, RPE-derived EVs also represent therapeutic vesicles for the treatment of inflammatory conditions including eye diseases. They are able to induce monocyte death or reprogram monocytes toward an anti-inflammatory phenotype (170).

## Conclusion

During recent decades, EVs have gained an emerging interest for both scientists and clinicians. Several studies have been conducted to obtain greater knowledge about the content of EVs, mechanisms of their biogenesis, functions, and involvement in diseases. As key players in pro- and anti-inflammatory events, EVs are of importance for the immune response and regulation (8, 42, 45). EV levels are enhanced in inflammatory diseases, particularly where complement activation occurs in a dysregulated manner, including polytrauma, TBI, hemorrhage, and septic shock (10, 36). The co-occurrence of EVs and complement indicate an important link in the context of innate immune responses. Current data gained from cellular experiments and clinical observations have confirmed that released EVs carry and modulate complement, and vice versa complement is also able to influence the number of circulating vesicles (Figure 3). Nevertheless, studies addressing this area of research are rare and thus, further scientific and clinical investigations are necessary for a deeper insight into the precise mechanistic interactions between complement and vesicles. In conclusion, EVs exhibit the potential as biomarkers and targets as well as therapeutic agents for complement and immunomodulatory strategies in the field of translational and personalized medicine, which are relevant in a broad scope of inflammatory diseases.

## Author Contributions

EK wrote the manuscript with the help from MH-L. All authors edited and commented on the manuscript. All authors read and approved the final manuscript.

## Conflict of Interest Statement

The authors declare that the research was conducted in the absence of any commercial or financial relationships that could be construed as a potential conflict of interest.

## Abbreviations

ALS, amyotrophic lateral sclerosis; AMD, age-related macular degeneration; AP, alternative pathway; APC, antigen-presenting cell; BBB, blood-brain-barrier; CARPA, complement activation related pseudoallergy; CCR5, C-C chemokine receptor type 5; CME, clathrin-mediated endocytosis; CNS, central nervous system; CP, classical pathway; CR1, complement receptor 1; CRP, C-reactive protein; DC, dendritic cell; EGFRvIII, epidermal growth factor receptor variant III; EV, extracellular vesicle; fMLP, N-formyl-methionyl-leucyl-phenylalanine; GPI, glycosylphosphatidylinositol; MAC, membrane attack complex; MARCKS, myristoylated alanine-rich C-kinase substrate; mCRP, monomeric CRP also neoepitope expressing CRP; MHC, major histocompatibility complex; MMP-2/-9, matrix metalloproteinase-2/-9; MV, microvesicle; MVB, multivesicular bodies; pCRP, native/pentameric CRP; pCRP*, neoepitope expressing CRP; PEG, polyethyleneglycol; PMN, polymorphonuclear leukocyte; PS, Phosphatidylserine; RPE, retinal pigmented epithelium; SNARE, soluble N-ethylmaleimide-sensitive fusion factor attachment protein receptor; TBI, traumatic brain injury; TF, Tissue factor.
